# DYSPHAGIA FOLLOWING HOSPITALIZATION IN NURSING HOME RESIDENTS WITH ALZHEIMER’S DISEASE AND RELATED DEMENTIAS

**DOI:** 10.1093/geroni/igac059.1075

**Published:** 2022-12-20

**Authors:** Nicole Rogus-Pulia, Raele Robison, Sweta Patel, Jennifer Bunker, James Rudolph, Joan Teno

**Affiliations:** University of Wisconsin-Madison, Madison, Wisconsin, United States; University of Wisconsin-Madison, Madison, Wisconsin, United States; Brown University, Providence, Rhode Island, United States; Oregon Health and Science University, Portland, Oregon, United States; Brown University, Providence, Rhode Island, United States; Brown University, Providence, Rhode Island, United States

## Abstract

Dysphagia frequently occurs in nursing home (NH) residents with Alzheimer’s Disease and Related Dementias (ADRD), often leading to serious health outcomes (e.g., pneumonia, malnutrition, reduced quality of life). While it is known that hospitalized NH residents with ADRD experience high rates of iatrogenic complications, dysphagia following discharge has not been examined. A retrospective cohort of all NH residents in the US (older adults aged ≥66) with advanced ADRD (Cognitive Function Scale ≥2), hospitalized between 2013-2017, and without a feeding tube or reported dysphagia on a Minimum Data Set (MDS) 3.0 assessment within 120 days prior to hospitalization was constructed. Treatment with intermittent mandatory ventilation (IMV) or non-invasive ventilation (NIV) during hospitalization and dysphagia status on the first post-hospitalization MDS was recorded. Data were analyzed using descriptive statistics and random effects multivariate logistic models that adjusted for age, gender, race/ethnicity, CFS score, ADL score, and comorbidities. Among the 805,199 residents with ADRD who survived the hospitalization and returned to the NH, new onset dysphagia occurred in 53,807 (6.7%; 95% CI 6.6-6.7) of residents. After adjustment, invasive mechanical ventilation (IMV) use was associated with increased risk of new onset of dysphagia (AOR 1.5; 95% CI 1.4-1.6) and non-invasive mechanical ventilation (NIMV) only slightly increased the risk (AOR 1.3; 95% 1.2-1.3). NH residents with ADRD are at risk for dysphagia following hospitalization. These findings emphasize the importance of swallowing evaluation and dysphagia treatment during hospitalization for ADRD patients, especially those treated with IMV or NIV, to prevent further negative health outcomes.

